# A Murine Point Mutation of Sgpl1 Skin Is Enriched With Vγ6 IL17-Producing Cell and Revealed With Hyperpigmentation After Imiquimod Treatment

**DOI:** 10.3389/fimmu.2022.728455

**Published:** 2022-06-13

**Authors:** Wenyi Yang, Binhui Zhou, Qi Liu, Taozhen Liu, Huijie Wang, Pei Zhang, Liaoxun Lu, Lichen Zhang, Fanghui Zhang, Rong Huang, Jitong Zhou, Tianzhu Chao, Yanrong Gu, Songhua Lee, Hui Wang, Yinming Liang, Le He

**Affiliations:** ^1^Henan Provincial Key Laboratory of Immunology and Targeted Therapy, School of Laboratory Medicine, Xinxiang Medical University, Xinxiang, China; ^2^Laboratory of Genetic Regulators in the Immune System, Henan Collaborative Innovation Center of Molecular Diagnosis and Laboratory Medicine, Xinxiang Medical University, Xinxiang, China; ^3^Laboratory of Mouse Genetics, Institute of Psychiatry and Neuroscience, Xinxiang Medical University, Xinxiang, China; ^4^Henan Collaborative Innovation Center of Molecular Diagnosis and Laboratory Medicine, School of Laboratory Medicine, Xinxiang Medical University, Xinxiang, China; ^5^CeleScreen SAS, Paris, France

**Keywords:** sgpl1, psoriasis, IL17, γδT cell, SPLIS

## Abstract

Sphingosine-1-phosphate lyase is encoded by the *Sgpl1* gene, degrades S1P, and is crucial for S1P homeostasis in animal models and humans. S1P lyase deficient patients suffer from adrenal insufficiency, severe lymphopenia, and skin disorders. In this study, we used random mutagenesis screening to identify a mouse line carrying a missense mutation of *Sgpl1* (M467K). This mutation caused similar pathologies as Sgpl1 knock-out mice in multiple organs, but greatly preserved its lifespan, which M467K mutation mice look normal under SPF conditions for over 40 weeks, in contrast, the knock-out mice live no more than 6 weeks. When treated with Imiquimod, *Sgpl1*^M467K^ mice experienced exacerbated skin inflammation, as revealed by aggravated acanthosis and orthokeratotic hyperkeratosis. We also demonstrated that the IL17a producing Vγ6^+^ cell was enriched in *Sgpl1*^M467K^ skin and caused severe pathology after imiquimod treatment. Interestingly, hyperchromic plaque occurred in the mutant mice one month after Imiquimod treatment but not in the controls, which resembled the skin disorder found in *Sgpl1* deficient patients. Therefore, our results demonstrate that *Sgpl1^M467K^* point mutation mice successfully modeled a human disease after being treated with Imiquimod. We also revealed a major subset of γδT cells in the skin, IL17 secreting Vγ6 T cells were augmented by *Sgpl1* deficiency and led to skin pathology. Therefore, we have, for the first time, linked the IL17a and γδT cells to SPL insufficiency.

## Introduction

The bioactive sphingolipid metabolite, sphingosine-1-phosphate (S1P), is commonly recognised as a major regulator of vascular and immune systems and could be relevant to skin function. S1P is biochemically derived from ceramide and irreversibly degraded by the intracellular enzyme sphingosine-1-phosphate lyase 1 (SPL, encoded by gene *Sgpl1*), which breaks down S1P into phosphoethanolamine and long-chain aldehyde. Previous studies have demonstrated that *Sgpl1* disruption can severely affect different organisms. For instance, *Sgpl1* deficiency in mice drastically elevates S1P, ceramide, and other sphingolipid concentrations, which affects the development of the kidney, brain, thymus, and other organs. Additionally, *Sgpl1* knock-out (or *Sgpl1*^−/−^) mice exhibit lymphopenia, anemia, and nephrosis, which limit their lifespan to no more than 6 weeks (1–4).

Likewise, the short-lived *Sgpl1*^−/−^ mice have prompted alternative experiment methods to study the role of SPL, including conditional knock-out or SPL inhibitor application (1, 2, 5, 6). In humans, SGPL1 deficiency, which is clinically referred to as SPL insufficiency syndrome (SPLIS) (7), is known to cause steroid-resistant nephrotic syndrome (SRNS), adrenal insufficiency, immunodeficiency, peripheral neurologic defects, and skin abnormality (8–13). In particular, SPLIS-caused skin abnormality is not well studied due to the lack of a proper mouse model (e.g., normal-lived). These abnormalities are often exhibited as hyperchromic plaque, acanthosis, and orthokeratotic hyperkeratosis in human patients (8, 9, 11–13). Therefore, the underlying molecular mechanisms of cutaneous immunity remain elusive.

Imiquimod, known as IMQ, is a TLR7 agonist. IMG induces psoriasis in mice, a widely used model for studying cutaneous immunology. IMQ stimulation activates DC and other innate immune cells, these innate immune cells recruit and prime IL17a producing γδT cells, namely γδ17T, within the treated skin. Moreover, these γδ17T cells are mainly composed of two populations: the Vγ4 (developed after birth) and embryonic thymocytes origin Vγ6 cells (14–16). The activated γδ17T produces massive IL17a and causes a psoriatic lesion, which manifests as demarcated, redness, scaly plaques, and thickened epidermis (17). Increased IL17a production promotes aberrant keratinocyte proliferation but blocks differentiation, resulting in cutaneous barrier disruption under inflammation (18, 19).

Forward genetic screening can facilitate the robust and unbiased identification of novel genes or alleles contributing to the function of immune cells (20). We performed ENU mutagenesis to identify genetic factors regulating T cell development and function in mice (21, 22). In the course of this work, we were surprised to discover that mice bearing the C57BL/6-*Sgpl1*^M467K Gris^ mutation in the *Sgpl1* locus (or *Sgpl1*^M467K^ as abbreviation), exhibited an acute severe cutaneous inflammation phenotype under IMQ treatment while also being dramatically longer-lived than the previously reported *Sgpl1* knockout mutant. Our results demonstrated that lesional skin of *Sgpl1*^M467K^ after recovery exhibits hyperchromic resembling hyperpigmented skin plaque in human SGPL1 disrupted patients. This finding may contribute new insights into how hyperpigmented plaque forms in SGPL1 disrupted human patients.

## Materials and Methods

### Mice and Genotyping

C57BL/6 mice were purchased from Beijing Vital River Laboratory Animal Technology Co., Ltd. *Sgpl1*^M467K^ mice were obtained by ENU mutagenesis on a C57BL/6 background. Its genotyping was confirmed by exome sequencing (raw data, **Supplement Tables S1****–****S5**; unprocessed sequencing data are available upon request), and the mice were used for study after being backcrossed with C57BL/6 for more than six generations. All mice experiments were performed according to the Xinxiang Medical University guidelines for animal care.

### Imiquimod-Induced Psoriasis Animal Model and IL17 Neutralization

Age-matched (6-10 weeks) C57BL/6 and *Sgpl1*^M467K^ were treated with Imiquimod cream (14 mg per ear, 5% Imiquimod) daily, for 6-10 days. The severity of the ear skin inflammation in mice was monitored and graded using the Psoriasis Area Severity Index (PASI). Erythema and scaling were scored independently on a scale from 0 to 4 (0, none; 1, slight; 2, moderate; 3, marked; and 4, very marked). The thickness of IMQ-painted ears was measured using a caliper (Kaefer) and scored according to the increase in thickness compared to day 0 (0, < 50um; 1, 50-100um; 2, 100-200um; 3, 200-250um; 4, >250um). The final value falls around 0-4, where 0 represents a slightly-changed ear thickness.

Regarding IL17a neutralization, anti IL17a antibodies (Cat#BE0173, BioXcell) or Isotype control antibodies (Cat#BE0083, BioXcell) were IV administrated at days -2, 1, 3, and 5 at 100ug/mouse; IMQ was painted on mouse ears from day 0 to day 6 (14mg/ear, 5% Imiquimod). The ear thickness, erythema, and scaling were monitored and scored as described before.

### Immune Cell Isolation and Antibodies

Lymph nodes and thymus were gently dissociated with a 2.5 syringe plunger on 100-um cell strainer (BD), the single-cell solution was collected and transferred to a 15mL tube (Falcon). Cells were counted per organ, and 2-4 M cells of each were used for staining. For ceramide measurement, LN cells were fixed with ice cold 4% formalin for 10 min (prepared with 1xPBS), after two times wash with 1xPBS, then subsequently stained with primary, surface antibodies mix, and secondary antibody mix. The skin immune cell extraction method was as described previously (23). The ear was separated into the dorsal and ventral layers, and two layers were further incubated in an HBSS digestion medium that contains 0.5mg/mL DNase (Cat# DN25, Sigma) and 0.25mg/mL Liberase (Cat#05401020001, Roche). After 90 min of incubation, the digested skin was further dissociated in gentleMACS™ C Tubes with Octo Dissociator (Miltenyi Biotec). All skin cells were used for staining. When IL17a was determined, the cells were first treated for 4h with 10ng/mL PMA (Cat#P1585, Sigma), 250ng/mL ionomycin (Cat#I9657, Sigma) and 0.7uL/mL BD GolgiStop (Cat#AB_2869012, BD Bioscience). Before staining, all cells were incubated with Fc Blocker for 20 min (2.4G2, homemade).

Cells were stained with antibodies and acquired with BD FACS Canto II (BD Bioscience) or Attune NxT Flow Cytometer before they were analyzed using the Flowjo 10.3 software. Antibodies used included: mouse anti-Ceramide (Cat#8104, Sigma), eflour450 conjugate anti-CD4(RM4-4), goat anti-mouse IgG (A-865), FITC conjugated anti-CD11b (CD3-12), anti-MHCII (M5/114.15.2), anti-CD3ϵ (145-2C11), anti-Vγ1(2.11); PE-conjugated anti-CD3ϵ (145-2C11), anti-CD44 (IM7), anti-Vγ4 (UC3-10A6); PerCP-eFluor710 conjugated anti- γ/δ TCR(eBioGL3); PerCP5.5 conjugated anti-CD4 (RM4-4); PE-Cy7 conjugated anti-IFNγ (XMG1.2), anti-CD11b(M1/70), anti-MHCii(M5/114.15.2), anti-TCRβ (H57-597); APC conjugated anti-CD5 (53-6.7), anti-IL17a (eBio17B7); Alexa Flour 700 conjugated anti-CD8a (53-6.7); Super Bright436 conjugated anti-CD3ϵ(500A2); eFluor506 conjugated Streptavidin; APC eFluor 780 conjugated anti-CD45 (30-F11), anti-CD24 (M1/69); biotin-conjugated anti- γ/δ TCR (UC7-13D5); except Brilliant violet 605 conjugated Streptavidin, PE-conjugated anti-Vγ4 (UC3-10A6), and FITC conjugated anti-Vγ1(2.11), all of which were purchased from Biolegend. All other antibodies were obtained from eBioscience.

### Western Blot

Freshly isolated mice kidney (0.1g) were homogenized in RIPA buffer (Cat#P0013B, Beyotime) containing 1x protease and phosphatase inhibitor cocktail (PhosSTOP™, Roche) using an overhead stirrer (IKA). Protein concentration was determined using the BCA method (Cat#P0012, Beyotime). Around 40 µg of protein was firstly separated by electrophoresis on a 10% polyacrylamide gel containing 0.4% sodium dodecyl sulfate (SDS), further electrophoretically transferred onto nitrocellulose membrane (Immobilon-P^SQ^, 0,2 µm, Millipore). Membrane was incubated in 5% fat-free milk in Tris-buffered saline with 0.1% Tween 20 (TBS-T) for 1 h at room temperature to block unspecific binding. The primary antibodies were used to detect SGPL1 protein (Cat#A15745, 1:1,000, ABclonal) and GAPDH (Cat#ab9485,1:3,000, Abcam). Horseradish peroxidase–conjugated secondary antibodies against rabbit (Cat#C31460100, Thermofisher) was used to detect the primary antibody.

### LC-MS

For sample preparation, the back skin was collected with the hair removed. The serum and skin tissue were homogenated and transferred into 1mL Methanol. After 30 min ultra-sonication, samples were centrifuged at 4°C, 12000rpm for 10 min. Afterward, 95uL of supernatant and 5uL Ceramide/Spingoid Internal Standard Mixture I (Avanti Polar Lipids) were mixed and prepared for measurement.

LC condition:

**Table:** 

LC system:	Waters ACQUITY UPLC I-CLASS.
Column(s):	Waters UPLC HSS T3 2.1 × 100 mm, 1.8 μm
Column temp.:	55°C
Flow rate:	0.26 mL/min
Mobile phase A (mpA):	6:4 Acetonitrile/water + 0.1% Formic acid +5 mM Ammonium acetate
Mobile phase B (mpB):	9:1 Acetonitrile/water + 0.1% Formic acid +5 mM Ammonium acetate
Gradient and run time:	0 min 68:32 mpA/mpB; 1.5 min 68:32 mpA/mpB; 15.5 min 15:85 mpA/mpB; 15.6 min 3:94 mpA/mpB; 18 min 3:97 mpA/mpB; 18.1 min 68:32 mpA/mpB; 20.0 min 68:32 mpA/mpB;
Injection volume:	4.0 µL

MS conditions:

**Table:** 

MS systems:	Xevo TQ-S micro
Ionization mode:	ESI (+)
Capillary voltage:	3.0 kV (+)
Source temp.:	150°C
Desolvation temp.:	350°C
Cone gas flow:	150 L/hr
Desolvation flow:	1000 L/hr

Informatics: the resulting data were processed with TargetLynx.

### Histopathology

Murine ear samples were embedded in paraffin and cut into 3 μm sections for tissue staining. Pathological observation was performed *via* H&E staining using light microscopy. Fontana-Masson staining was applied for Melanin staining. The melanin quantification method has been described previously (24). First, we randomly chose the ear section and used the ImageJ software, and manually selected the epidermis to highlight the stained melanin content *via* automatic segmentation. The melanin content is represented as the total pixels within the selected region; the mean gray value is calculated by dividing the total pixels of melanin content by the selected area.

### Isolation of RNA and qPCR

Total RNA was extracted from the ear skin lesion using an RNeasy Plus Mini Kit according to the manufacturer’s instructions (Qiagen). Total RNA (300 ng) was used for cDNA synthesis with a MEGAshortscript™ T7 Transcription Kit (Thermo Fisher Scientific). qRT-PCR, based on TB Green Premix Ex Taq™ (TaKaRa), was performed to quantify mRNA levels on a BIO-RAD CFX Connect Real-Time System (CFX-96). The value of 2^-ΔCt^ was used to determine the fold changes between samples. The sequences of the primers used throughout this study were: TNFα (Forward (F), 5’-GGTGCCTATGTCTCAGCCTC-3’; Revers (R), 5’-ACTGATGAGAGGGAGGCCAT-3’), IL-17 (F, 5’-GAAGGCCCTCAGACTACCTCAA-3’; R, 5’-CAGCTTTCCCTCCGCATTGAC-3’), IFNγ (F, 5’-GCGTCATTGAATCACACCTG-3’; R, 5’-TGAGCTCATTGAATGCTTGG-3’), IL-10 (F, 5’-GCTCTTACTGACTGGCATGAG-3’; R, 5’-CGCAGCTCTAGGAGCATGTG-3’), IL-23 (F, 5’-TATCCAGTGTGAAGATGGTTGTG-3’; R, 5’-CACTAAGGGCTCAGTCAGAGTTG-3’), Vγ6 (F, 5’-TGCTACAAGTCTTCACACTGGC-3’; R, 5’-CATCGGGCTTCTGAACACTTGT -3’), Sgpl1 (F, 5’- CTGAAGGACTTCGAGCCTTATTT-3’; R, 5’- ACTCCACGCAATGAGCTGC-3’); HPRT (F, 5’-CGTCGTGATTAGCGATGATG-3’; R, 5’-ACAGAGGGCCACAATGTGAT-3’).

## Results

### C57BL/6 Mice With Sgpl1^M467K^ Point Mutation Permits Modeling of Human Skin Pathology in SPL Insufficiency Syndrome

Skin disorders, including the exhibition of scaly hyperchromic plaques, are repetitively observed in SPLIS syndrome, which is caused by SGPL1 deficiency. Its underlying mechanisms were previously relatively unknown. In this study, we obtained an *Sgpl1* mutant through ENU mutagenesis in mice on the C57BL/6 background to study SPLIS related skin abnormalities.

**Figure 1:**
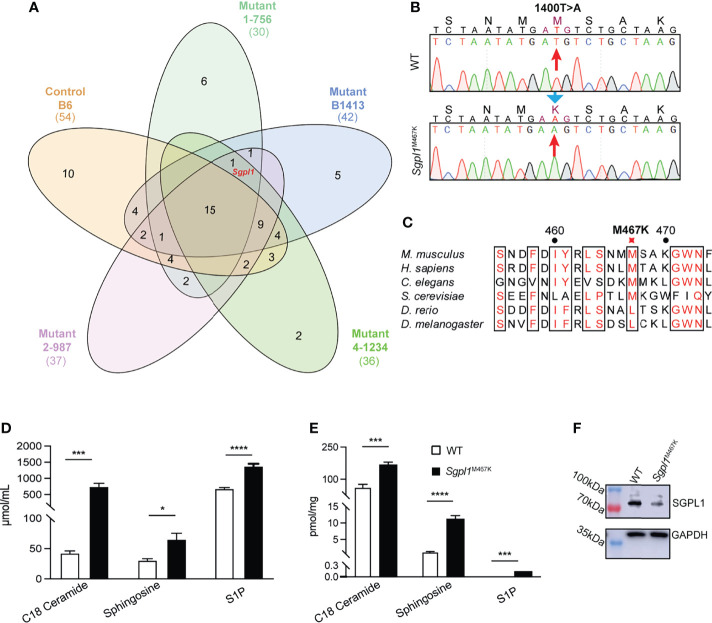
Characterization of *Sgpl1*^M467K^ mutation. **(A)** Venn diagram of the shared genes with homozygous mutation between different mice. Numbers represent the number of shared genes among different mice. Red marked Sgpl1 gene is the only gene which is in all four lymphopenia mice of the same colony (1-756, B1413, 4-1234, 2-987) but absent from the B6 gene list (**Supplement Tables S1–S5**). **(B)** Mononucleotide mutation was identified and localized on 1400 (Ref. NM_009163), T changed to A. **(C)** Multiple protein sequence alignment of 6 Sgpl1 proteins from Mus musculus and the homologues, including *Mus musculus* (NP_033189.2), *Homo sapiens* (NP_003892.2), *Caenorhabditis elegans* (NP_505372.1), *Drosophila melanogaster* (NP_652032.1), *Danio rerio* (XP_005156777.1), *Saccharomyces cerevisiae* (NP_010580.1). **(D, E)** Comparison of serum **(D)** and cutaneous **(E)** C18-Ceramide, Sphingosine, S1P concentration of wild type and *Sgpl1*^M467K^ mice; five wild-type and five *Sgpl1*^M467K^ mice were analyzed, please note S1P concentration of wild type was under detection threshold and was defined as 0 during statistic evaluation; original TIC within **Supplementary Table S6**. *, p<0.05; ***, p<0.001; ****, p<0.0001; unpaired t-test, error bar represents s.e.m. **(F)** Western blot analysis of SGPL1 expression in kidney of wild type and Sgpl1^M467K^ mice; experiment repeated twice; full-length pictures were shown in **Supplementary Figure S1A**.

We got the mutant line by FACS screening of peripheral white blood cells. The mouse colony exhibited with lymphopenia phenotype was established after back crossed to B6 for more than six generations. We confirmed the mutant phenotype was only co-segregating with a mutation on *Sgpl1* gene *via* exon sequencing on four different mutant mice of the same colony and B6 control (**Figure 1A**). The same method has also been described in previous studies (21, 22). This mutant line carries a missense mutation on *Sgpl1*, and its SPL protein changed methionine to lysine at position 467 (Ref. NP_033189.2) by thymine to adenine transversion (**Figure 1B**). We call this mutation “C57BL/6-*Sgpl1*^M467K Gris^” (or *Sgpl1*^M467K^). Comparative genome analysis revealed that the amino acid of SPL at the 467 position is conserved from worms to humans (**Figure 1C**).

**Figure 2:**
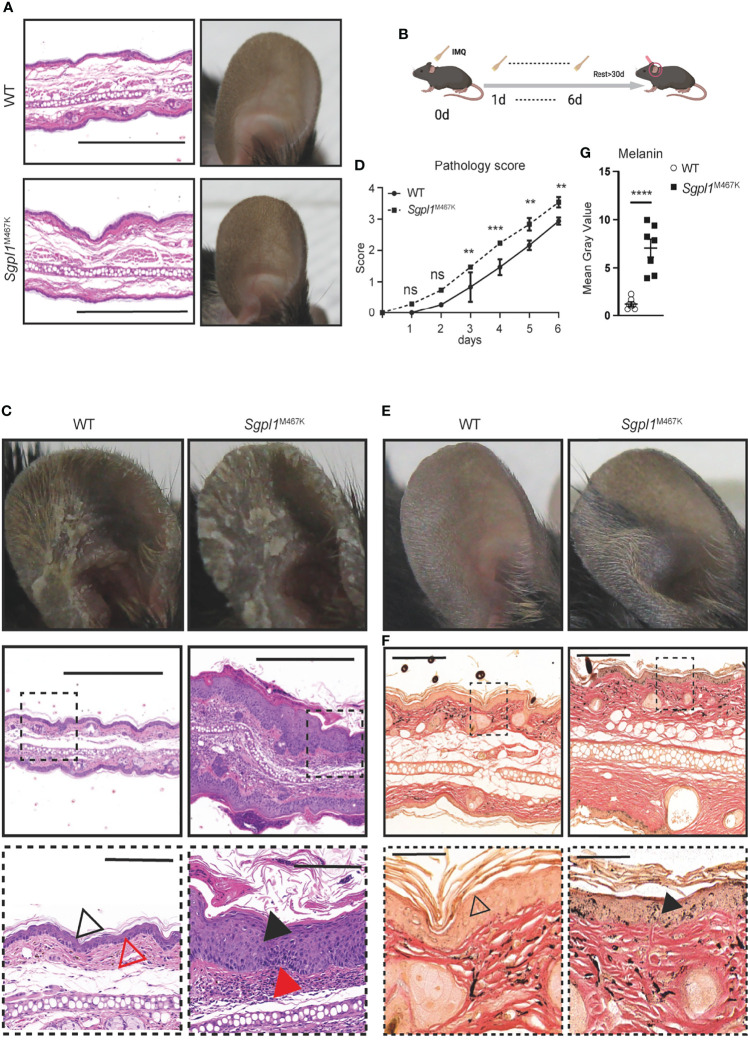
*Sgpl1*^M467K^ mice exhibit severe cutaneous pathology after IMQ treatment. **(A)** Ear images of WT and *Sgpl1*^M467K^ mice in steady-state. Left, H&E staining, scale bar, 500µm; right, ear photo. Representative image from ≥3 mice of each genotype. **(B)** Experiment flow chart for IMQ application; IMQ was applied on the mouse ear from day 0 to day 6 and the thickness, erythema, and scaling score of the treated ear were recorded each day; ear hyperpigmentation was checked after more than 30 days. **(C)** Ear images of WT and *Sgpl1*^M467K^ mice at day 7 of IMQ induction. Top, ear photo; middle and bottom images, H&E stained; bottom images were enlarged from square marked middle regions, middle and bottom images scale bars represent 500µm and 100µm, separately; black triangle, epidermis, and acanthosis; red triangle, the dermis. Representative images were depicted from ≥ 6 ears per genotype. **(D)** The pathological pattern was determined by ear thickness, erythema, and scaling score of each experimental day (for each category please refer to **Supplement Figure S1**, details see *Materials and Methods*). Experiments were repeated five times with three mice from each genotype, one was chosen as representative. ****, p<0.0001; ***, p<0.001; **, p<0.01; ns, not significant; analyzed with two-way ANOVA. **(E, F)** Representative ear images, 30 days IMQ-treatment cessation; **(E)** ear photo. **(F)** Fontana-Masson stained ear section; bottom images were enlarged from top ones, triangle pointed to epidermal melanin. Representative images from ≥ 6 ears per genotype. **(G)** Melanin content was assessed from the epidermal region of seven randomly selected independent Fontana-Masson stained sections (see *Materials and Methods*). ****,p<0.0001, unpaired t-test, error bar represents s.e.m.

It is important to note that such *Sgpl1*^M467K^ mice can live up to 40 weeks (**Supplement Figures S1E–G**), while the *Sgpl1*^−/−^ mice have a short lifespan limited to a maximum of six weeks (4). Consistent with what has been observed in other *Sgpl1* deficiency mouse models (3, 5), the S1P and its precursors, ceramide (C18), sphingosine concentration were significantly higher in *Sgpl1*^M467K^ skin and serum compared to wild type; moreover, the S1P concentration was around 0.14 pmol/mg in mutant skin, while it was under detectable threshold within the wild-type skin (**Figures 1D, E**). Therefore, it is not surprising that *Sgpl1*^M467K^ mice experienced kidney failure and increased bone formation (**Supplement Figures S1A, D, E, F, G**), the same as knock-out mice (1, 25). These mutant mice did not exhibit visible skin alterations under steady-state; their ear anatomy and thickness remained the same as the wild-type mice (**Figure 2A**; **Supplement Figure S1H**. The SGPL1 protein level is decreased in *Sgpl1*^M467K^ mice kidneys (**Figure 1F**). However, its mRNA expression was not affected by the M467K mutation (**Supplement Figures S1B, C**).

We used Imiquimod (IMQ) to induce inflammation in the skin and compared the pathological changes between *Sgpl1*^M467K^ mice and the wild-type controls (**Figure 2B**). We observed elevated skin pathology on the ear of *Sgpl1^M467K^* mutants compared to the wild-type control during the early stages of IMQ application. Pathological assessment by H&E staining demonstrated that mutant ears had excessive acanthosis and hyperkeratosis and that the cornified layer in mutants was thicker and largely detached from the epidermis (**Figure 2C**). We quantified the pathology grade each day and found significantly higher in mutant mice from day 3 to day 6 than in the wild-type control (**Figure 2D**; **Supplement Figure S2**). Our *Sgpl1*^M467K^ mutant model allows for the long-term study of skin after IMQ application since it has an extended lifespan compared to knock-out mice. 4 weeks after ending the IMQ treatment, we observed hyperpigmentation on IMQ-treated mutant ears compared to wild-type mice (**Figure 2E**). Interestingly, such hyperchromic alterations have occurred in SPLIS patients (8, 9, 12, 13). The skin sections of recovered IMQ-treated *Sgpl1*^M467K^ mice stained with Fontana-Masson showed markedly increased levels of melanin within the epidermis when compared to their wild-type counterparts (**Figures 2F, G**). In this study, we identified a novel mutant model that can model skin disorders in SPLIS patients.

### Sgpl1^M467K^ Mutation Severely Impairs αβ T Cell Development

**Figure 3:**
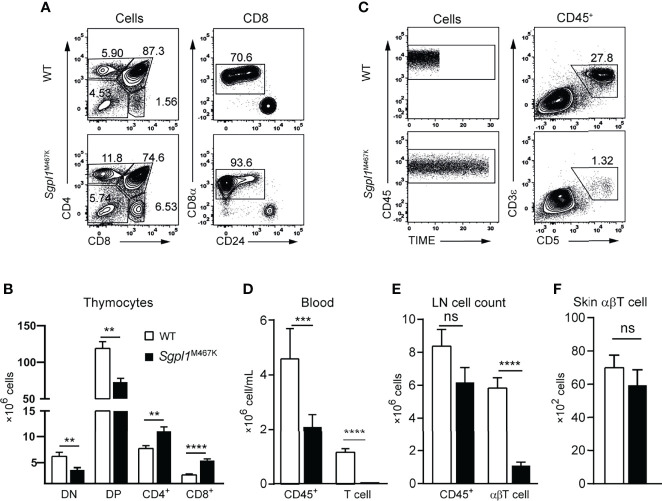
*Sgpl1*^M467K^ mutation impaired αβT cell development and cell count in blood and LN. **(A, B)** Thymocytes were analyzed with a flow cytometer. **(A)** Representative flow cytometer figure; the DN, DP, CD4 subsets were defined from all cells; the CD8^+^ was further defined from CD8 single-positive cells by removing the ISP population. **(B)** Absolute number of different thymocytes subsets within a thymus. Data were pooled from sixteen mice from four different experiments. **(C, D)** Periphery blood lymphocytes and T cell count were accessed with Attune Flow cytometry. **(C)** Representative flow cytometer figure; the same amount of CD45^+^ cells were acquired using the same flow speed, the different length of time under the same arbitrary unit reflects the CD45^+^ cell concentration. **(D)** Blood cell concentration. Experiments repeated more than 10 times, data was from fourteen mice of one representative experiment. **(E)** Absolute cell counts of LN CD45^+^ and abT cells. Data pooled from eighteen mice from three independent experiments. **(F)** Skin αβT count under steady-state. Data pooled from two experiments of thirteen mice. ****, p<0.0001; ***, p<0.001; **, p<0,01; ns, not significant; analyzed with unpaired t-test, error bar represents s.e.m.

Considering that excessive inflammation due to αβT cells can contribute to the exacerbated skin pathology of *Sgpl1*^M467K^ mutant mice after IMQ treatment, we analyzed whether αβT cell development was affected by point mutations. Consistent with previous reports using *Sgpl1*^−/−^ mice (15), we found that the number of CD4^+^ or CD8^+^ single-positive thymocytes was significantly increased in *Sgpl1*^M467K^ mice, suggesting that single-positive thymocytes were sequestered within the thymus. In contrast, double-positive (DP) and double-negative (DN) cell count significantly decreased (**Figures 3A, B**), which indicates impaired thymic αβT cell development. The blood of *Sgpl1*^M467K^ mutant mice exhibited a 2-fold reduction of the absolute CD45^+^ white blood cells. Notably, there were drastic T cell reductions in the peripheral blood, and the *Sgpl1*^M467K^ mutant T cell experienced a 37-fold decrease (**Figures 3C, D**). Similarly, we observed that the number of αβT cells decreased 4-fold in the lymph nodes (**Figure 3E**). Ceramide, the chemical precursor of S1P, its concentration was elevated in *Sgpl1* knockout mice and induced αβT cell apoptosis (2, 3, 26, 27). Correspondingly, we found cellular ceramide was highly accumulated in *Sgpl1* ^M467K^ αβT cells (**Supplement Figures S3A, B**); the mutant αβT cell also demonstrated a poor survival rate in recipient mice after adoptive transfers (**Supplement Figures S4A–C**). Moreover, we found that the mutant cutaneous αβT cell count was similar to the wild type counterpart under steady-state (**Figure 3F**).

### Sgpl1^M467K^ Mice Exhibit Elevated Cutaneous γδT Cell Count After IMQ Treatment

**Figure 4:**
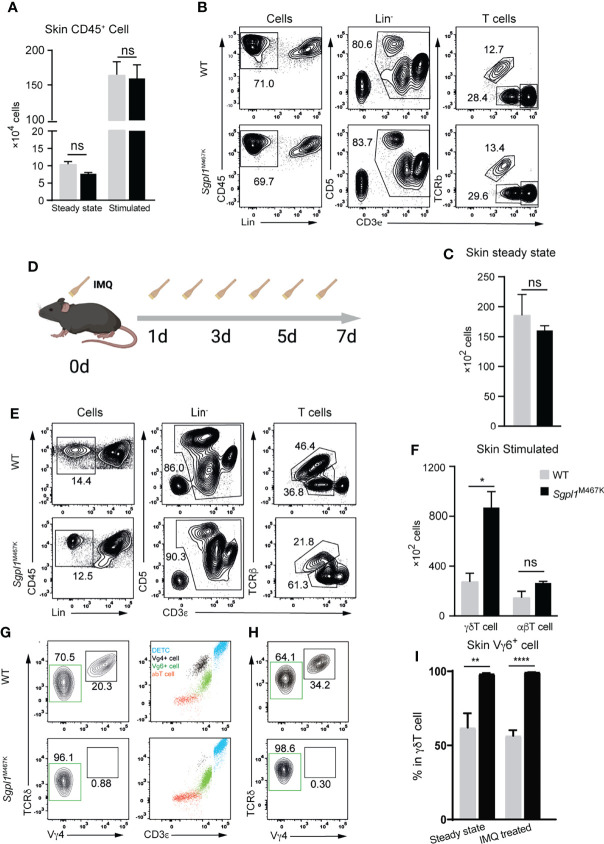
*Sgpl1*^M467K^ skin exhibited elevated VγδT cell count after IMQ treatment. **(A)** Total white blood cell count within steady-state or inflamed skin (two ears). **(B, C)** T cell count in steady-state skin determined by flow cytometer. **(B)** Representative flow cytometer figure, T cells were grouped from the Lin^-^(not CD11b^-^ or MHCii^-^) cells by CD3^+^, which was further divided into TCRβ^+^ αβT cells and TCRβ^-^ CD3^medium^ γδT cells and CD3^hi^ dendritic epidermal T cells (DETC). **(C)** γδT cell count of two ears. **(D–F)** Skin T cell count after IMQ treatment. **(D)** Experiment flow chart for IMQ application, mouse ears were treated with IMQ from day 0 to day 6. **(E)** Representative flow cytometer figure, the same as **(B)**; **(F)** absolute T cell count after IMQ stimulation. **(G–I)** Vγ4 Vγ6 γδT cell subsets within the steady-state or inflamed ear. **(G)** Representative flow cytometer figure of steady-state skin; left, Vγ4 or Vγ6 subpopulation composition of skin γδT cells; right, blue dots are CD3^hi^ γδT^hi^ DETC, red dots are γδT^-^ CD3^low^ αβT, black dots are CD3^medium^ γδT^medium^ Vγ4^+^ T cells, green dots are Vγ4^-^ CD3^bright^ γδT^low^ Vγ6 T cells. **(H)** Representative flow cytometer figure of Vγ4, Vγ6 cells within inflamed skin. Representative figure of steady-state skin data were displayed from one of three independent experiments, which analyzed thirteen wild-type mice and eleven mutant mice; inflamed skin data is from two independent experiments, which analyzed six wild-type mice and six mutants; steady-state ear Vγ4 Vγ6 distribution data were pooled from two experiments, which analyzed eight wild-type and six mutant mice; inflamed ear Vγ4, Vγ6 distribution data displayed are from one experiment, which analyzed four wild-type and four mutant mice; ****, p<0.0001; **, p<0.01; *, p<0,05; ns, not significant; unpaired t-test.

Considering that skin αβT cell count was not affected by *Sgpl1*^M467K^ mutation, we investigated cutaneous immune cells before and after IMQ treatment. We found that the CD45^+^ white blood cell counts and γδT were the same between *Sgpl1*^M467K^ mice and wild-type mice under steady-state (**Figures 4A–C**). After seven days of IMQ treatment (**Figures 4D**), both control and mutant mice experienced a ~20-fold increase of CD45^+^ white blood cells in the ear skin (**Figures 4A, B, E**). Even though the IMQ treated *Sgpl1*^M467K^ skin was demonstrated with severe pathology, the neutrophile percentage remained the same between the wild-type and mutant, in contrast to its increase in *Sgpl1*^M467K^ spleen and ear draining LN (**Supplement Figure S3D**). Intriguingly, *Sgpl1*^M467K^ mutant skin γδT cell count was dramatically increased under IMQ stimulation and 2-fold more γδT cells were found in the inflamed mutant ears compared to the IMQ treated WT control (**Figures 4E, F**).

Previous studies have demonstrated that Vγ4^+^ and Vγ6^+^ γδT cell subsets in the skin are the leading IL17 producers in the IMQ-induced psoriasis mouse model (14, 28). Therefore, we sought to understand the involvement of both cell types within the mutant skin. Since there is no commercially available Vγ6 antibody, we used the CD3^bright^ γδT feature (29) to gate Vγ6^+^ subset (**Figure 4G**). Surprisingly, *Sgpl1*^M467K^ mutation greatly affected the composition of cutaneous γδT subsets in steady-state and under inflammation. The mutant skin is enriched with nearly 99% Vγ6^+^ T cells in steady-state, with almost no Vγ4^+^ T cells. In contrast, the wild-type cutaneous γδT cells are composed of 60% Vγ6^+^ and 40% of Vγ4^+^ cells (**Figure 4G**). This situation remained the same after IMQ treatment: the Vγ6^+^ subset is still the virtually only population of mutant skin γδT cells and comprised only 60% of the wild-type counterpart (**Figure 4H, I**). Correspondingly, quantitative PCR analysis on Vγ6 expression was higher in IMQ-induced *Sgpl1*^M467K^ psoriatic lesion compared to the wild type (**Supplement Figure S5E**). Moreover, we also observed elevated CD44 expression on *Sgpl1* skin γδT cells (**Supplement Figure S5F**); CD44 serves as an activation marker for T cells (30).

The αβT cell count remained the same between the wild type and the *Sgpl1*^M467K^ before and after IMQ treatment (**Figures 4B, E, F**, **Figure 3F**). The decreased level of S1P receptor 1 expression on LN αβT cells compared to LN Vγ4 T cells (**Supplement Figures S4D, E**) may indicate that part of the αβT cell can avoid the S1P gradient dependence and enter the skin. Additionally, we observed that the *Sgpl1*^M467K^ γδT cell was outnumbered in peripheral LN and the spleen in competitive chimeric bone marrow transfer experiments compared to its wild-type competitors. This indicates that γδT cell accumulation could benefit from cell-intrinsic *Sgpl1*^M467K^ mutations (**Supplement Figures S5A–D**). Our findings demonstrate that *Sgpl1*^M467K^ mutation promoted Vγ6^+^ γδT cell counts in IMQ-induced inflamed lesion and significantly impaired cutaneous Vγ4 γδT cell population.

### Induction of IL17a by Elevated γδ 17T Led to Severer Psoriatic Pathology in Sgpl1^M467K^ Skin

**Figure 5:**
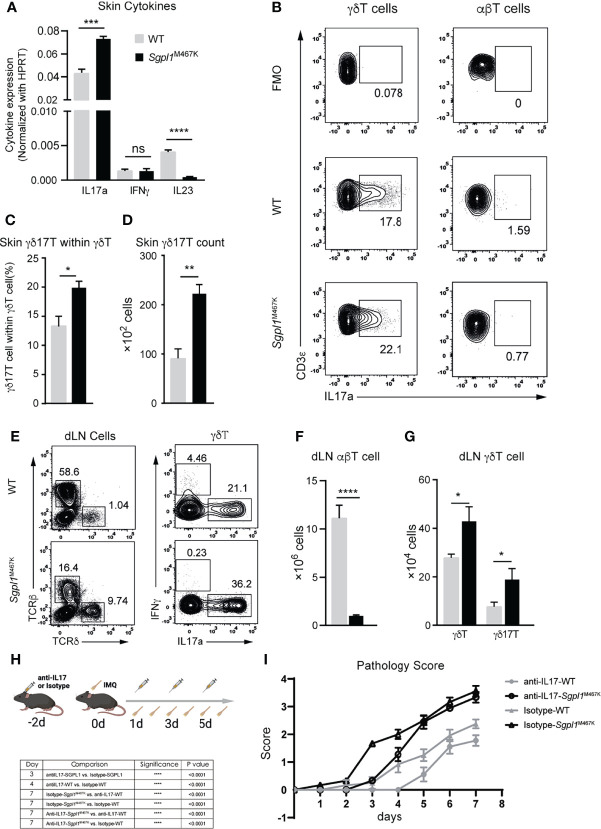
Elevated levels of IL17a^+^ cells contributed to IMQ-induced acute pathology in *Sgpl1*^M467K^ skin lesions. **(A)** Cytokine expression within psoriatic lesions was measured with quantitative PCR 7 days after IMQ treatment. The experiment was conducted two times. **(B–D)** Lesional γδ17T count after 7 days after IMQ induction was determined with flow cytometer; **(b)** representative flow cytometer figure for γδ7T cell or IL17^+^ αβT cell population. **(C)** γδ17T proportion within the γδT population; **(D)** γδ17T cell counts within the skin lesion. **(E–G)** Draining LN cell count after 6 days of IMQ treatment determined by flow cytometer. **(E)** Representative flow cytometer figure; left, total draining LN cells were sub-grouped to αβT cells and γδT cells; right, γδT cells were tested for IFNγ or IL17a expression. **(F, G)** αβT cell, γδT, and γδ17T cell count within draining LN. For both skin and its draining LN analysis, data were pooled from two experiments, which analyzed five wild-type and six mutants; ****, p<0.0001; ***, p<0.001; **, p<0.01; *, p<0.05; ns, not significant; unpaired t-test. **(H, I)** IL17 neutralization postponed pathology progression on IMQ treated *Sgpl1*^M467K^ skin; **(H)** experiment flow-chart, IL17 antibodies or Isotype antibodies were administrated at days -2, 1,3, and 5; IMQ was painted on mouse ears from day 0 to day 6; **(I)** Pathology score was determined by ear thickness, erythema, and scaling score from day 0 to day 7; the statistical analysis is listed on the left table; similar results were obtained from two repeated experiments; analyzed with two-way ANOVA.

Previous studies have found that γδ17T and IL17a are the primary executors for acute IMQ-induced inflammation (18, 19, 31). To further elucidate the specific type of immune response resulting from IMQ-treatment on *Sgpl1*^M467K^, we used qRT-PCR to verify the expression of IL23, IL17a, and IFNγ in skin lesions on day seven of IMQ stimulation. Of these cytokines, only IL17a was significantly expressed, while IL23 was significantly repressed (p-value < 0.0005) in *Sgpl1*^M467K^ skin compared to the wild type. No difference was observed for IFNγ (**Figure 5A**).

Highly produced IL17a suggested the presence of γδ17T in a psoriatic lesion, leading us to speculate that γδT (but not αβT) was involved in our model based on the observed enriched γδT cell count in *Sgpl1*^M467K^ lesional skin. As expected, the IL17a-expressing γδ17T cell population was found with a more significant proportion in *Sgpl1*^M467K^ skin lesion (**Figures 5B–D**). In contrast, the IL17a^+^ αβT cell was merely visible (**Figure 5B**). Regarding the ear draining LN, we observed that *Sgpl1*^M467K^ had elevated γδT and γδ17T cell counts compared to the wild type (**Figures 5E, G**). However, the αβT cell count was significantly lower in ear draining LN of *Sgpl1*^M467K^ than the wild type (**Figures 5E, F**).

To provide direct evidence that IL17a contributes to acute psoriatic pathology, the mice were treated with antiIL17a neutralising antibodies or isotype IgG control antibodies (100ug per mouse *via* intravenous injection (IV)) as illustrated (**Figure 5H**). The administration of antiIL17 antibodies delayed the progression of the pathology of *Sgpl1*^M467K^ mutant mice; the mutant group treated with antiIL17 antibodies had a lower pathology score (0.3, day 3) compared to the mutant mice treated with isotype antibodies (1.7, P<0.0001). As a reference, similar phenomena were observed on wild-type mice on day 4 between the group treated with antiIL17a neutralising antibodies and the group treated with isotype IgG antibodies. After all, the mutant group showed severe pathology after day 5, regardless of IL17 neutralisation (**Figure 5I**). These results indicate that excessive production of IL17a by γδ17T cells in *Sgpl1*^M467K^ skin lesion contributed to its acute psoriatic pathology.

### Sgpl1^M467K^ Mutation Impairs Thymic Vγ4 and Vγ1 γδT Cell Emigration but Not Cutaneous Vγ6 and DETC

**Figure 6:**
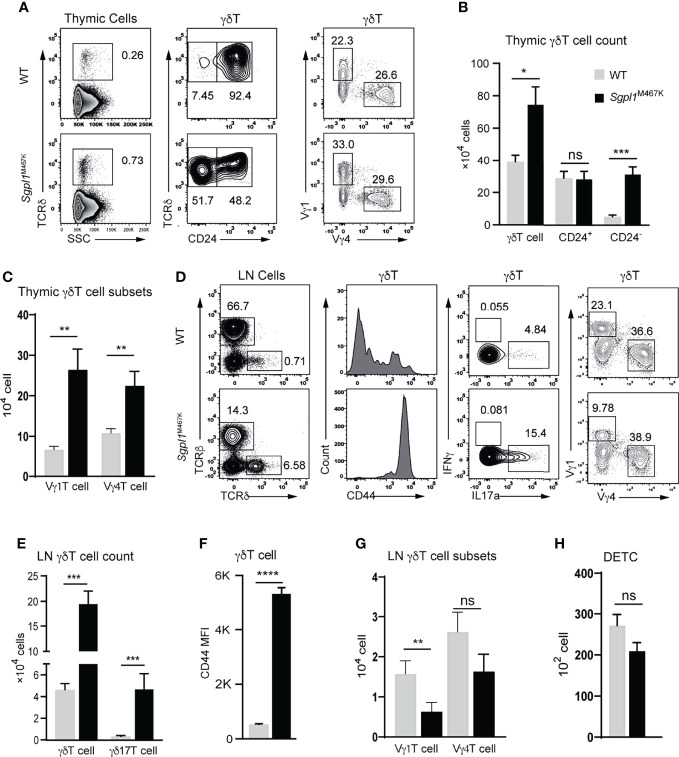
*Sgpl1*^M467K^ mutation partially impairs γδT cell migration. **(A–C)** Thymic γδT cell count was accessed with flow cytometer. **(A)** Representative flow cytometer figure; left, γδT cells were grouped from total thymocytes; middle, the γδT cell were subdivided into CD24^+^ and CD24^-^ populations, or Vγ1 and Vγ4 populations on the right. **(B, C)** Absolute cell count of thymic γδT cell population; data pooled from three experiments **(B)**; data pooled from three experiments, which analyzed seven wild-type and five mutant mice. **(D–G)** γδT cells counts within mesenteric LN (mLN) were accessed with a flow cytometer. **(D)** Representative flow cytometer figure; left, γδT cells were grouped from total mLN cells; the CD44 expression, or IL17a^+^, IFNγ^+^, Vγ4^+^, Vγ1^+^ populations were analyzed. **(E)** γδT and γδ17T cell count. **(F)** γδT cell CD44 mean fluorescent density (MFI). **(G)** Vγ4^+^ or Vγ1^+^ cell count within mLN. Data were from two experiments, three-four mice were used for each experiment. **(H)** DETC count from two ears; gating strategy refers to **Figure 3B**. Data pooled from thirteen wild-type and eleven mutant mice. ****, p<0.0001; ***, p<0.001; **, p<0.01; *, p<0.05; ns, not significant; unpaired t-test.

Given that *Sgpl1*^M467K^ mutation impaired αβT cell thymic development but promoted γδ17T inflammation in the skin, we investigated what happened to the development of γδT cells during the steady-state for *Sgpl1*^M467K^ mice. When we quantified the γδT cell number in the thymus, we observed a significant increase of thymic γδT cells in *Sgpl1*^M467K^ compared to wild type (**Figures 6A, B**). CD24 expression defines thymic progenitor γδT cell during development (32), leading us to use it to study thymic γδT cell development. Interestingly, the immature CD24^+^ γδT count in *Sgpl1*^M467K^ remained the same with the wild type but matured CD24^-^ γδT cells were 20-fold higher in the mutant (P-value < 0.0001, **Figures 6A, B**). Likewise, the thymic Vγ1^+^ and Vγ4^+^ cell counts were approximately two times and one time higher than the wild type, respectively (**Figures 6A, C**). These results demonstrate that *Sgpl1*^M467K^ mutation blocked the exit of the γδT cell from the thymus, which has been observed for αβT cells in our point mutation mice (**Figures 3A, B**) and Sgpl1 knock-out mice (3). However, this did not affect γδT cell maturation in the thymus.

LN serves as a source of immune cells for the adjacent tissue, which leading us to explore the status of LN γδT cells in a steady state. First, we observed that the number of LN γδT and γδ17T cells in *Sgpl1*^M467K^ significantly increased compared to the wild-type (P-value < 0.001, **Figures 6D, E**). CD44 is a marker of γδ17T cells in LN (33) and was dominantly expressed in *Sgpl1*^M467K^ LN γδT cells compared to wild type (**Figures 6D, F**). There were fewer Vγ1 cells in mutant LN, though Vγ4 cell counts were not affected compared to the wild type (**Figures 6D, G**).

Like cutaneous Vγ6 cell count, the other important murine skin γδT subset DETC was not affected by the *Sgpl1*^M467^ mutation. Its cell count was similar in mutant and wild-type skin (**Figure 6H**). In line with skin Vγ4 cell data, we also observed diminished Vγ4 cells in *Sgpl1*^M467K^ mutant lungs, livers and intestines (**Supplement Figure S6**).

Our findings reveal that the *Sgpl1*^M467K^ mutation did not affect γδT cell development in the thymus but blocked its emigration from the thymus. While Sgpl1 mutation impacted γδT subsets differently in LN, it also blocked Vγ4 migration from draining LN to its adjacent tissue since these cells are absent in the skin but sufficient in LN.

## Discussion

We have successfully identified a previously undescribed *Sgpl1* mutant mouse strain *via* ENU mutagenesis. It enabled us to study the impact of *Sgpl1* deficiency in adulthood, which was not possible by using the *Sgpl1*^−/−^ mouse. We used an Imiquimod-induced inflammation experiment model to explore the augmented immune response on *Sgp1l*^M467K^ skin. Our *Sgpl1*^M467K^ strain notably displays a severe cutaneous γδT-IL17 inflammation response upon IMQ treatment. However, regardless of IMQ treatment, the prominent IL17 producer Vγ6 cell comprised 99% of the γδT cells in *Sgpl1*^M467K^ skin, in contrast to the 60% of Vγ6^+^ cells within the wild type. Notably, Th17 cells (CD4^+^ IL17^+^ cell) only trivially changed upon IMQ stimulation. It has been demonstrated that *Sgpl1* disruption promoted liver-associated Th17-IL17 inflammation (34). Studies have proven that Th17 cells play a critical role in autoimmunity (19). In the context of *Sgpl1* mutated mice, the relevance between elevated IL17 inflammation and Th17 or γδ17T could be tissue or organ-dependent. Additionally, γδ17T cells are abundant in mouse liver, which was not investigated in this study (34).

Our results demonstrate the formation of hyperpigmentation plaque on skin lesions in an *Sgpl1* deficient mouse model for the first time. We postulated that the rapid induction of IL17a during inflammation contributed to the formation of hyperpigmentation plaque on skin lesions. Currently, anti-inflammatory monoclonal antibody therapies, such as anti-IL17a, have been used to effectively treat psoriasis patients (35). However, this often causes hyperchromic skin on the recovered lesion (36). Elevated cytokine levels have been reported in psoriatic plaque, including IL17a, which induce massive melanocytes proliferation but prohibit melanogenesis (37). Once IL17a production stopped or ablated by anti-IL17a monoclonal antibody, melanocyte retrieves their melanin-producing capacity and skin lesion formed hyperpigmentation rapidly (38, 39). This indicates that hyperchromic plaque manifested in SGPL1 deficient patients could reflect their biased IL17 related pro-inflammatory response in the cutaneous immune system.

We found that *Sgpl1*^M467K^ skin developed acute pathology during early IMQ treatment. However, the role of *Sgpl1* in skin psoriasis pathology was unclear, particularly its S1P lyse enzymatic activity. It has been suggested that S1P promotes keratinocyte differentiation. Therefore, *Sgpl1* inhibition or S1P administration can attenuate hyperkeratosis during psoriasis vulgaris progression and serve as a putative method of treating this disease (6, 40–42). Nevertheless, evidence from tamoxifen-inducible *Sgpl1* knock-out mice has shown *Sgpl1* malfunctions boosted S1P but diminished C16 ceramide concentration within the skin and induced hyperkeratosis, which threatened *Sgpl1* inhibition for psoriasis treatment (5). Though, the pathology on these tamoxifen-induced Sgpl1 knock-out mice was very mild compared to ours: the H&E skin section presented as hyperkeratosis was only revealed with two layers of epidermal keratinocytes (5) it is the figure of the cited paper (**Figure 1D**). Our Sgpl1^M467K^ mice were demonstrated with elevated cutaneous S1P and C18 ceramide concentration but were not showing any skin abnormality in steady-state. These contradictory conclusions reflect the complex role of sphingolipids on different cells in the skin. On one side it induces keratinocyte differentiation and attenuates skin pathology. On the other side, these metabolites can also serve as a stimulator for inflammatory immune responses. As Lipid serves as a potential human γδT cell antigen, and murine Vγ6 cells regulate lipid metabolism in adipose tissue (43, 44). These pieces of evidence suggest the *Sgpl1* deficiency may promote Vγ6 cell activation by supplying Vγ6 T cells with a potent antigen within the elevated S1P lipid precursors once the antigen-presenting cells were activated by imiquimod. As IL17 neutralization was delayed but did not block the pathology of IMQ treated *Sgpl1*^M467K^ mice. There should be other unexplored links between psoriasis pathology and *Sgpl1* deficiency.

SPLIS patients also experienced neurological defects, such as microcephaly, progressive ptosis, and ulnar nerve paralysis et al. As a sphingolipid-rich organ, the brain relies on *Sgpl1* for its enzymatic function. Developmental neural-targeted *Sgpl1* ablation (*Sgpl1*^flox/flox/NES^ mouse) caused massive S1P accumulation in the brain, altered presynaptic architecture, and induced cognitive deficits (45). Though *Sgpl1* deficiency caused neural defects that are partially attributed to the metabolic reagent, its underlying mechanism is still unclear (46), making it important to explore the immune factors involved in this neuronal symptom. The meninges cover central nerve systems and provide a supportive framework for the brain and spinal cord. Like the skin, there is a sizeable Vγ6T cell population residing within the meningeal, which has recently been recognized as an important factor for controlling the central nervous system development and inflammation (47, 48). After considering *Sgpl1* deficiency-induced γδ17T cutaneous inflammation, it is next necessary to verify the role of meningeal γδT cells in neuronal disorders of SPLIS patients. As conditional *Sgpl1* depletion cannot represent global *Sgpl1* deficiency in SPLIS patients, *Sgpl1*^M467K^ mice serves as a better model for studying SPLIS.

It has been reported that the Sgpl1 knockout or conditional knockout have elevated levels of thymic mature αβT cells (2, 3, 49). Intriguingly, the same phenotype can be achieved by dendritic cell-intrinsic *Sgpl1* deficiency. This discovery indicated thymic DC are responsible for maintaining the S1P gradient between the thymus and peripheral circulation (49). Our unpublished data on wild type mice which reconstituted with *Sgpl1*^M467K^ bone marrow provides supplementary evidence by showing diminished blood lymphocytes in recipient mice. In line with this evidence, the *Sgpl1*^M467K^ mutation greatly impacted γδT thymic emigration. Therefore, it is extremely interesting to know that *Sgpl1*^M467K^ skin is enriched with Vγ6, DETC and αβT cells, but is absent with Vγ4. Systematic studies have demonstrated that Vγ6 cells and DETCs develop from the embryonic thymus and the Vγ4 cells are from the post-natal stage (14, 50, 51). The mating cage for producing *Sgpl1*^M467K^ mice was maintained with female heterozygotes and male homozygotes, and the heterozygous maintains a normal S1P environment. It is possible the *Sgpl1*^M467K^ Vγ6 or DETC cells can resident to the skin normally compared to the Vγ4. The different emerging times for Vγ6, Vγ4 and DETC during development may also suggest that Vγ6 cells and DETC can easily exit from the unclosed embryonic thymus-circulation barrier (52) and enter other tissues such as the skin. In contrast, Vγ4 cells were confined by dysregulated S1P gradient and a tight barrier in the adult thymus and LN. In LN, Vγ4 cells expressed a higher level of S1PR1 compared to αβT cells which indicates S1P may play a more important role in these cells. The αβT cells may enter the *Sgpl1*^M467K^ skin in an S1P independent way, or these cells are like Vγ6 cell and DETC developed in the early development stage and thus were not restricted by the *Sgpl1* deficiency. It is also possible that the Vγ6 cells or DETC do not rely on S1P signalling for thymic emigration. Considering the importance of IL17a producing γδT cells on the skin and other autoimmune diseases (53), it is crucial to elucidate the underlying molecular mechanism behind how *Sgpl1* deficiency affects γδ17T cell subsets differently. A better understanding of these concepts could allow for the future use of γδ17T cells to treat cutaneous infections or other diseases (54).

## Data Availability Statement

The raw data supporting the conclusions of this article will be made available by the authors, without undue reservation.

## Ethics Statement

The animal study was reviewed and approved by Committee on animal care at Xinxiang Medical University.

## Author Contributions

Conceptualization: LH, YL, and HW; Data curation and formal analysis: LH, WY, BZ, QL, LT, PZ, LL, LZ, FZ, RH, JZ, SL, TC, and YG; Writing - original draft preparation: LH, YL, and HW. All authors contributed to the article and approved the submitted version.

## Funding

This work was supported by the National Natural Science Foundation of China (grant n° 81901573 to LH, grants n° 81471595 to YL, grants n° 32000491 to BZ, grants n° U1904157 to LL).

## Conflict of Interest

The authors declare that the research was conducted in the absence of any commercial or financial relationships that could be construed as a potential conflict of interest.

## Publisher’s Note

All claims expressed in this article are solely those of the authors and do not necessarily represent those of their affiliated organizations, or those of the publisher, the editors and the reviewers. Any product that may be evaluated in this article, or claim that may be made by its manufacturer, is not guaranteed or endorsed by the publisher.
