# Supplementary material for: A Murine Point Mutation of Sgpl1 Skin Is Enriched With Vγ6 IL17-Producing Cell and Revealed With Hyperpigmentation After Imiquimod Treatment
Source: Front Immunol. 2022 Jun 13;13:728455. doi: 10.3389/fimmu.2022.728455 (PMC9234551; doi:10.3389/fimmu.2022.728455)
Supplement: Supplementary file 7 [file Presentation_1.pdf]

Supplement Figure1

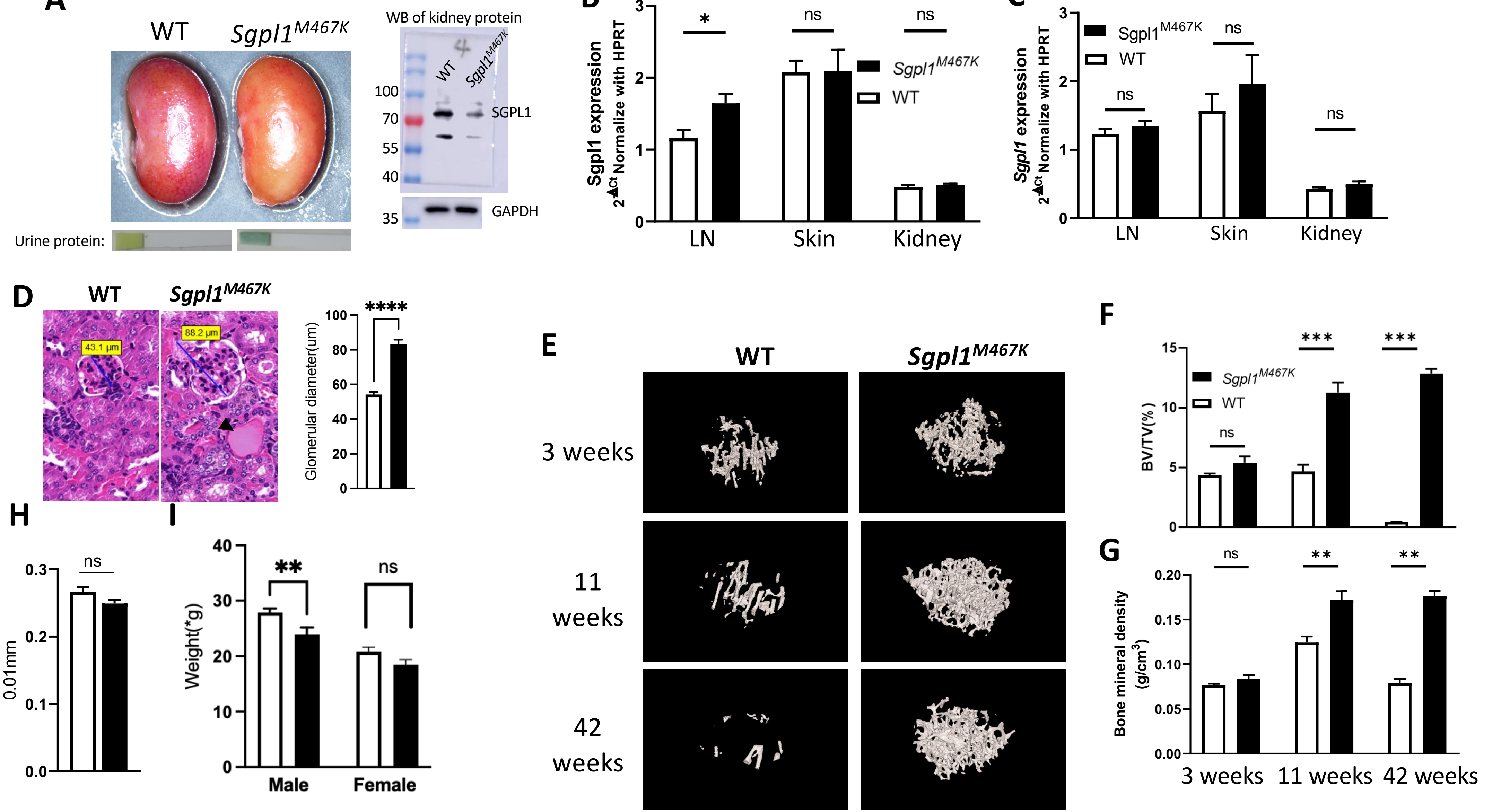

Supplementary Figure 1. *Sgpl1*^{M467K} mutation and its multiple organs deficiency phenotype. (A) Kidney of 9 weeks mice; bottom, fresh urine was analyzed with protein test strips (URIT 1V, URIT Medical Electronic Co., Ltd); urine protein of wildtype mice was less than 15, *Sgpl1*^{M467K} urine was more than 300 according to the manufacture's indication; uncropped full-length pictures of western blotting membranes presented in the main Fig. 1 e., membrane was cut after 40kDa to enable blotting of anti-SGPL1 and anti-GAPDH antibody. (B,C) *Sgpl1* expression level in mice LN, skin and Kidney; Samples of 2 WT and 2 mutant mice; (B) primer pair 1, forward primer linked Exon2 and Exon3 (5'-CTGAAGGACTTCGAGCCTTATTT-3'), revers primer located on exon3 (5'- ACTCCACGCAATGAGCTGC-3'); experiment repeated also with other samples, similar results obtained; (C) primer pair 2, forward primer linked Exon4 and Exon5 (5'-ACGTAAGATCGAACAACAGGTG-3'), revers primer located on Exon 5 (5'-GCTGTGCCCATACCCTGAG-3'). (D) Representative kidney H&E staining image of 9 weeks mice and glomerulus diameter analysis, black arrow pointed to the protein cast. (E,F,G) µCT analysis on the bone trabecular with Burker SkyScan1276, under 55kV source voltage, 200uA source current, 6um image pixel size, 0.3° rotation step, AI 0.25mm filter. (E) the representative image of trabecular from different ages mice; (F,G) the percentage of BV/TV and mineral density of the trabecular. Data for 3 weeks mice were from 8 WT and 6 mutant samples; 11 weeks, 4 WT and 4 mutant samples; 42 weeks, 2 WT and 2 mutant samples. (H) The ear thickness under steady state; data were pooled from 12 WT and 12 mutant mouse. (I) Weight of adult mice, male: 26 WT, 15 mutant, female: 15 WT, 14 mutant. ****,$p<0.0001$, ***, $p<0.001$;*,$p<0.05$; ns, not significant; unpaired t test.

Supplement Figure2

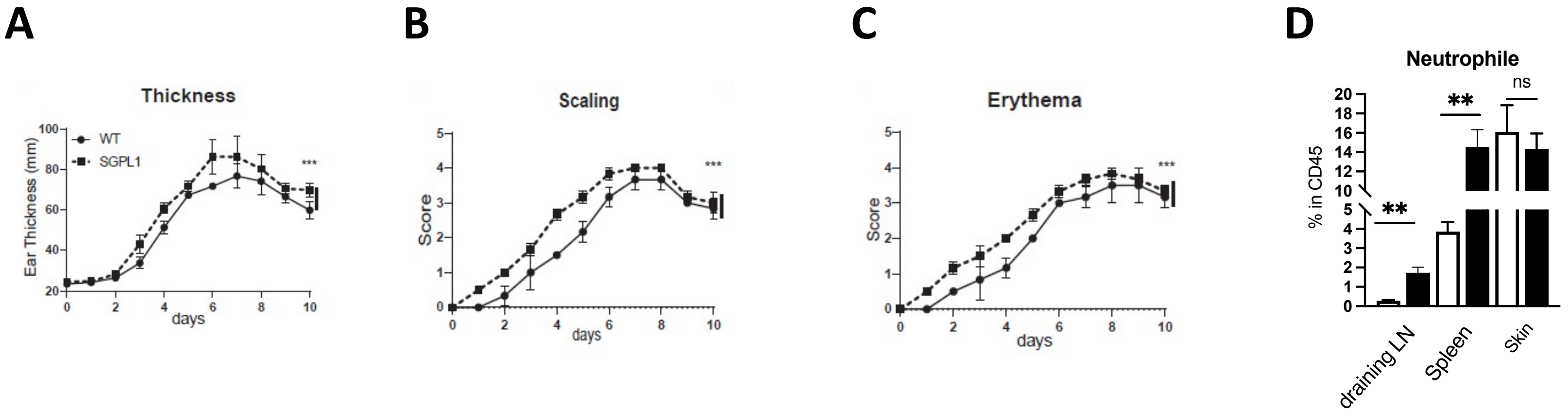

Supplement Figure 2. Pathological record and neutrophile proportion of IMQ treated mouse. (A,B,C)IMQ was applied on mouse ear for 10 constitutive days; pathological patterns: ear thickness, erythema and scaling were determined each experimental day. Pathology evaluation method was described before (Terhorst et al., 2015). ***, $p<0.0003$; paired t test. (D) Neutrophile proportion within CD45+ white blood cells in draining LN spleen and skin after 6 days of imiquimod treatment. *, $p<0.05$; ns, not significant, unpaired t-test.

Supplement Figure3

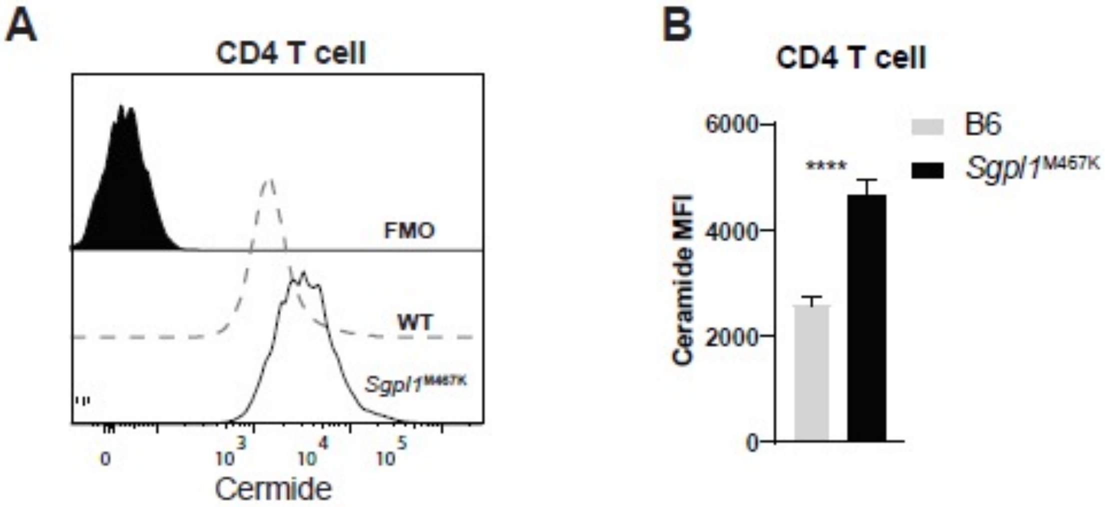

Supplementary Figure 3. *Sgpl1*^{M467K} LN CD4^{+} $\alpha\beta$T cells is with higher ceramide content. (A) Representative flow cytometer figure; ice cold 4% formalin(prepared with 1xPBS) fixed LN cells were stained with anti-CD4 eflour450 (Cat#48-0041-82, ebioscience) and ant-Ceramide (Cat#C8104,Sigma) and anti-mouse IgG APC (Cat#A-865, eBioscience), further analyzed with BD Canto II and Flowjo software. (B) Ceramide MFI. Experiment repeated for more than 3 times, each time with 6-10 samples, one representative depicted. ****, p<0.0001; unpaired t test.

Supplement Figure4

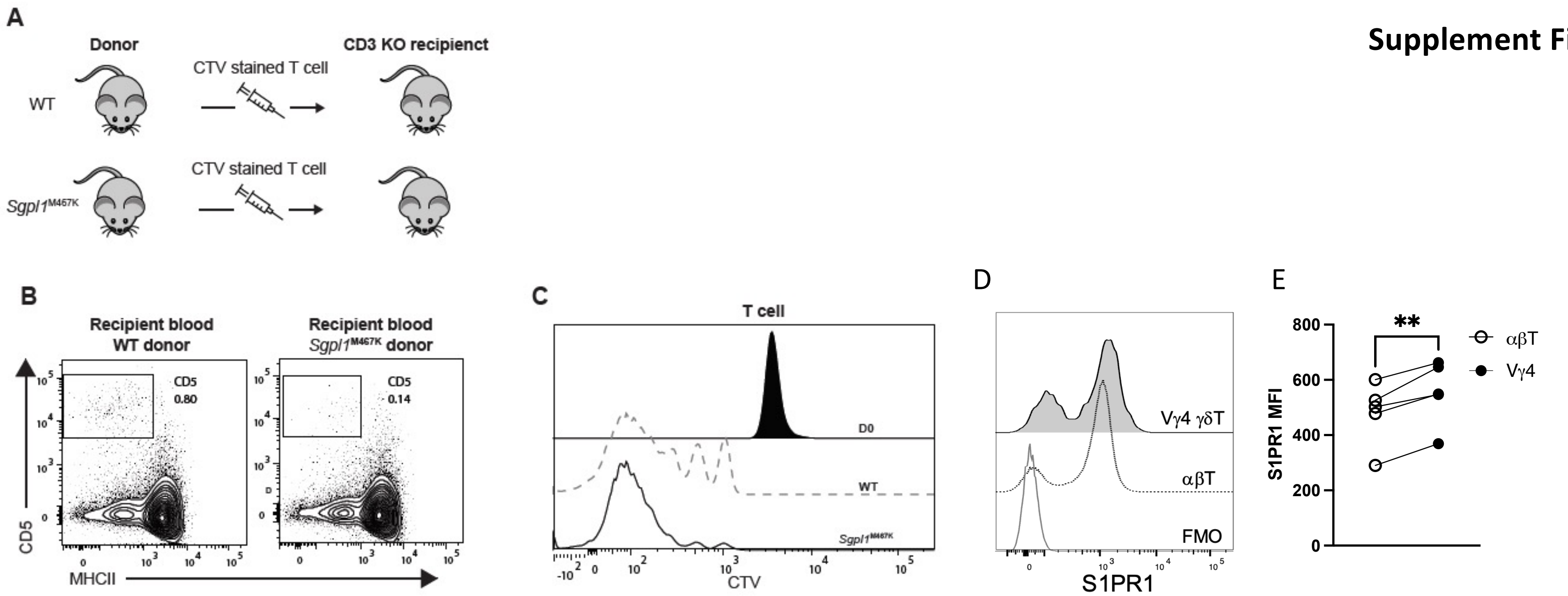

Supplementary Figure 4. *Sgpl1*^{M467K} $\alpha\beta$T cell poorly survived in adaptive transferred recipient.

(A) Experiment skim; the purified T cells from wild type or Sgpl1M467K mutant mouse were firstly stained with CTV, and then injected into the CD3 KO recipient; 5 days later the LN cells of the recipient mice were analyzed for CTV dilution. (B,C) Representative flow cytometer figure of recipient LN cells. Cells were stained with anti-CD5 PEcy7 (Cat#25-0051-81, eBioscience), anti-MHCii (Cat#E10058-1636,eBioscience); cells analyzed with BD Canto II, data analyzed with flowJo software. (C) Donor $\alpha\beta$T cells within recipient LN, concatenated from 3 mice for each genotype. Experiment performed with 3 mice of each genotype. (D) Representative flow cytometer figure for LN Vg4 gdT cell and abT cell S1P receptor 1 (S1PR1) mean fluorescent. (E) S1PR1 MFI. Experiments repeated for two times, 1 representative depicted, 5 wildtype mice were tested. **, $p<0.01$; ns, not significant; unpaired t test.

Supplement Figure5

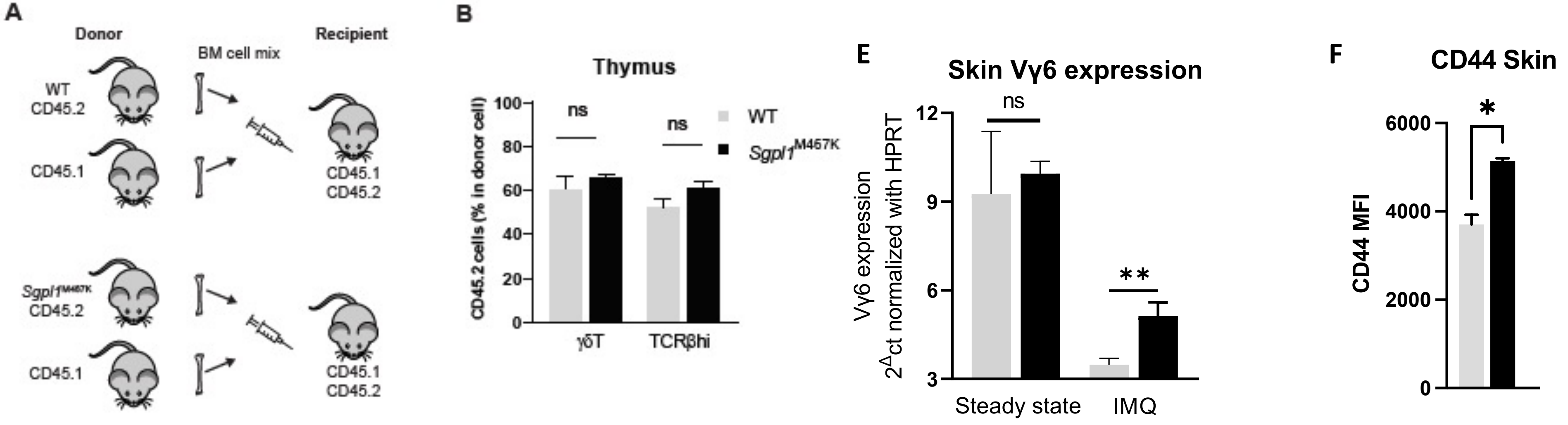

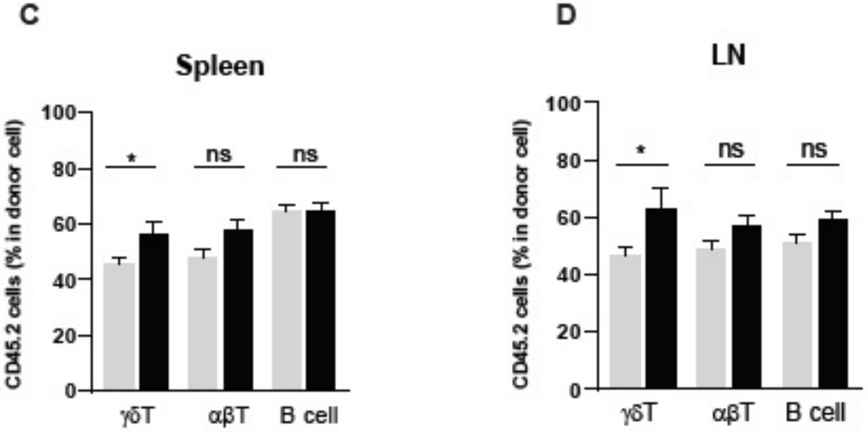

Supplementary Figure 5. Peripheral *Sgpl1*^{M467K} γδT cell is slightly more competitive than wild type γδT cell in chimeric recipient.

(A) Experiment skim; the CD45.1^{+} wild type mouse bone marrow were mixed with CD45.2^{+} wild type or Sgpl1M467K mutant mouse bone marrow in a ratio of 1:1; 4 M of mixed bone marrow were injected into the Busulfan (Cat#B2635, Sigma) conditioned CD45.1^{+} CD45.2^{+} recipient; 2 months later, recipient mice thymus, spleen and LN were analyzed for cell composition. (B-D) Proportion of CD45.2 cell within the sum of donor CD45.2 and CD45.1 cells of different organs. 10 recipient were analyzed. (E) Skin Vg6 expression within steady state skin and psoriatic lesion (IMQ treated day 6) were measured with quantitative PCR. 2 WT and 2 mutant skin tissue were analyzed under both conditions. (F) CD44 MFI of γδT cell in wildtype or mutant skin treated with IMQ (IMQ treated day 6). **, p<0.01; *, p<0.05; ns, not significant; unpaired t test.

Supplement Figure6

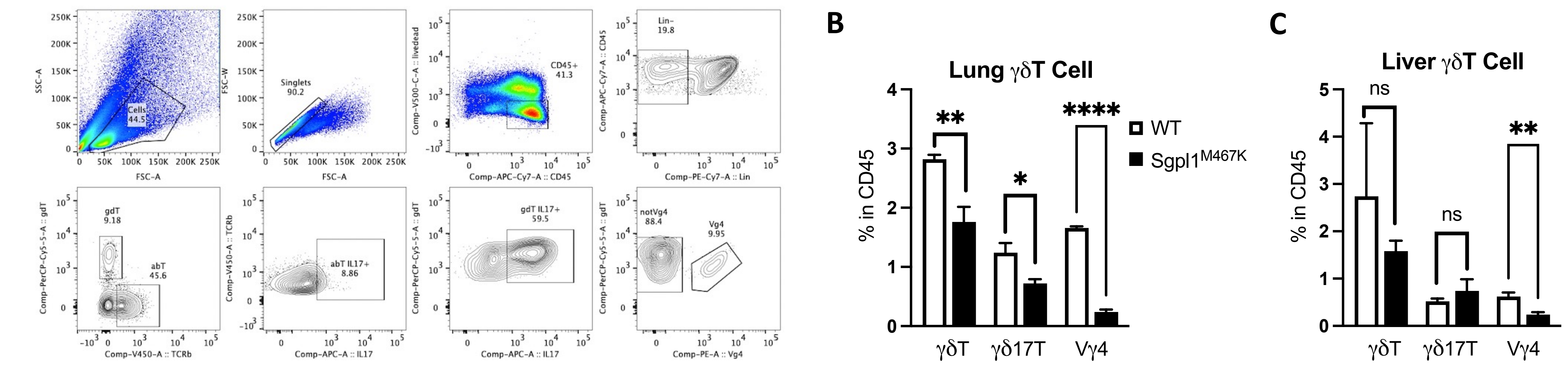

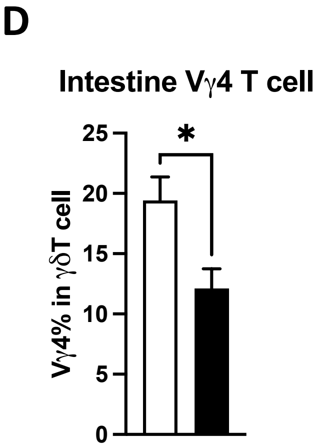

Supplementary Figure 6. γδT cell in Lung, Liver and instestine.
(A) Flow cytometer analysis, cell gateing strategy. (B-D) Proportion of γδT cell or Vγ4 T within lung (B), liver (C), and intestine (D). Regarding liver and lung, experiments repeated two times, one depicted, 5 WT and 5 mutant were analyzed under both conditions. Intestine repeat for 4 times, with 4 wt and 4 mutant, all samples were summed. ****, $p<0.0001$; **, $p<0.01$; *, $p<0.05$; ns, not significant; unpaired t test.

Materia and methods

Urine protein test

A drop of fresh urine was dropped onto protein test strips (URIT 1V, URIT Medical Electronic Co., Ltd), 20s later the protein value was read according to the manufactures indication panel.

µCT analysis

For µCT analysis the femur was obtained and fixed in 4% paraformaldehyde. The bone trabecular with scanned with SkyScan1276 (Burker, Germany), under 55kV source voltage, 200uA source current, 6um image pixel size, 0.3° rotation step, Al 0.25mm filter.

T cell adoptive transfer

We followed the protocol as described before (Wang, 08). Briefly, the wild type or Sgpl1M467K mutant mouse LN cells were firstly purified with Dynabeads purification kit (Cat#1413D, Thermofisher), then stained with CellTrace Violet (CTV) cell proliferation kit (Cat#C34557, Thermofisher). The stained CTV cells were intravenous injected into the CD3 KO recipient (2M/mouse); 5 days later the LN cells of the recipient mice were analyzed for CTV dilution with flow cytometer.

Bone marrow chimeric analysis

We followed the protocol described previously(Peake, 15) to prepare the competitive bone marrow chimeric mice. Briefly, the CD45.1+ CD45.2+ recipient mice were intraperitoneal injected with 30mg/kg/day busulfan(Cat#B2635, Sigma) with one interval, until a total dose of 90mg/kg/mouse reached. Two days after the last busulfan administration, the recipient mouse were injected i.v with bone marrow mix. For bone marrow mix preparation, the CD45.1^{+} wild type mouse bone marrow were mixed with CD45.2^{+} wild type or Sgpl1M467K mutant mouse bone marrow in a ratio of 1:1; 4 M of mixed bone marrow were injected into the Busulfan conditioned CD45.1^{+} CD45.2^{+} recipient; 2 months later, recipients' thymus, spleen and LN were analyzed for cell composition.

Protein structure in silico modeling

Both wild type and M467K mutation protein structure were predicted by the SWISS-MODEL (https://swissmodel.expasy.org/). The protein morphology and hydrogen bone were further analyzed with PyMOL software.

Wang Y, Kissenpfennig A, Mingueneau M, Richelme S, Perrin P, Chevrier S, et al. Th2 Lymphoproliferative Disorder of Lat Y136F Mutant Mice Unfolds Independently of TCR-MHC Engagement and Is Insensitive to the Action of Foxp3 ⁺Regulatory T Cells. J. Immunol. 2008;180:1565–75

Peake K, Manning J, Lewis C-AA, Barr C, Rossi F, Krieger C. Busulfan as a Myelosuppressive Agent for Generating Stable High-level Bone Marrow Chimerism in Mice. J. Vis. Exp. Journal of Visualized Experiments; 2015;2015(98):1–7
